# The treatment of follicular lymphoma with CD19-directed chimeric antigen receptor T-cell therapy

**DOI:** 10.3389/fonc.2024.1384600

**Published:** 2024-06-05

**Authors:** Ryan Jacobs, Caron Jacobson

**Affiliations:** ^1^ Levine Cancer Institute, Charlotte, NC, United States; ^2^ Dana–Farber Cancer Institute, Boston, MA, United States

**Keywords:** lymphoma, follicular lymphoma, CAR T, axicabtagene ciloleucel, tisagenlecleucel (tisa-cel, Kymriah), lisocabtagene maraleucel, mosunetuzumab

## Abstract

Follicular lymphoma (FL) is the most common indolent non-Hodgkin lymphoma. Significant unmet need remains for patients with relapsed/refractory FL after ≥3 lines of prior therapy. While recent advancements have likely improved the survival of patients with FL, most patients will eventually relapse. The treatment of patients with FL after multiple relapses or those with refractory disease has historically led to lower overall response rates (ORR) and shorter progression-free survival (PFS) with each subsequent line of therapy. New treatments with high ORR and durable PFS are needed in this setting, particularly in patients that progress within 2 years of first line chemoimmunotherapy (POD24) and/or those refractory chemoimmunotherapy. Chimeric antigen receptor T-cell therapies targeting the B-cell antigen CD-19 have shown to be an efficacious treatment option for both heavily pretreated patients and/or patients with refractory FL, resulting in a high ORR and durable remissions.

## Introduction

In the United States, follicular lymphoma (FL) is the most common indolent non-Hodgkin lymphoma (NHL), accounting for approximately 35% of NHLs, and has an estimated incidence of 3.18 cases per 100,000 people (1). Numerous risk factors have been purported to be linked to FL, but none have been validated, and the incidence has been stable over time. FL has increased prevalence in white populations where the incidence is more than twice that in African and Asian populations (2). The incidence of FL increases with age with the median age at diagnosis being 65 years (3).

Significant breakthroughs have been made in the treatment of FL in recent decades. Effective frontline treatment with chemoimmunotherapy involving anti-CD20 antibodies has led to durable remissions in most patients (4, 5). However, about 20%–25% of patients will have significantly shorter progression-free survival (PFS) and early progression after chemoimmunotherapy within the first 2 years from initial treatment (POD24). These patients have been shown to have poor long-term outcomes and reduced overall survival (OS) (6). Additionally, the treatment of relapsed/refractory (R/R) FL after ≥2 lines of prior therapy is associated with progressive shortening of PFS with each line of treatment (7–9). The approval of new classes of drugs including immunomodulatory agents and epigenetic modulators has improved outcomes for patients with multiple R/R FL, but these patients continue to represent an unmet need (10).

Recently, three novel therapies that engage T cells have been approved by the Food and Drug Association (FDA) and have been incorporated into the treatment armamentarium of patients with FL with R/R disease. They include CD20/CD3-bispecific antibody mosunetuzumab and the CD19-directed chimeric antigen receptor (CAR) T-cell therapies axicabtagene ciloleucel (axi-cel) and tisagenlecleucel (tisa-cel) (11, 12). We will further discuss the commercial use of axi-cel and tisa-cel as well as review the available data on an additional CD19-directed CAR T-cell therapy lisocabtagene maraleucel (liso-cel) in the treatment of patients with R/R FL. Additionally, we will contrast the use of CAR T-cell therapy with mosunetuzumab as well as review some initial data of new investigational CAR T-cell technologies.

## Early studies in CD19 CAR T cells used in the treatment of FL

Targeting CD19 with CAR T-cell therapy was initially explored in lymphoma in R/R diffuse large B-cell lymphoma (DLBCL), where it revolutionized the natural history of chemorefractory disease and, as a result, changed the treatment paradigm and outlook for these patients (13–15). As CD19 is widely expressed in B-cell malignancies, assessing the efficacy in high-risk multiple relapsed FL, where no standard therapy was established, was likewise investigated. Initial reports of activity in R/R FL came out of early-phase studies from the National Cancer Institute investigating the use of a CD19-targeted CAR T-cell product with a CD28 costimulatory domain that would become axi-cel. In an early report of activity seen in a patient with FL, Kochenderfer et al. described dramatic lymphoma regression and noted that B-cell precursors were selectively eliminated from the patient’s bone marrow for approximately 39 weeks. The targeted and prolonged elimination of B-lineage cells indicated eradication of CD19+ B cells that were antigen-specific and that the adoptive transfer of anti–CD19-CAR T cells could be a promising approach for treating B-cell malignancies like FL (16). Longer-term follow-up confirmed activity in a larger number of patients with FL, where a 3-year duration of response (DOR) was 63% for the eight patients treated with low-grade lymphoma on this study (17, 18).

Tisa-cel was originally developed from CTL019, whose activity in FL was first reported by Schuster et al., where 15 patients with R/R FL were treated with CTL019 with high overall response rate (ORR) and a Complete Remission (CR) rate of 71% (19). Median peak expansion of the CTLO19 cells appeared to occur later (median of 8 days) than the CD28 co-stimulated axi-cel, with a more gradual expansion of CAR T cells over time but increased area under the curve (20). A 5-year update was later reported on these patient and was encouraging, with a 5-year PFS of 43% and median DOR having not been reached (21).

Hirayama et al. later reported the results from the Fred Hutchinson Cancer Research Center where eight patients with R/R FL were treated on a phase I/II trial with CD19 CAR T cells on a 1:1 ratio of CD4/CD8+ T cells and the co-stimulatory molecule 4-1BB that would later become liso-cel (22). The reported ORR was high, and the majority of the patients with FL treated (88%) obtained a CR. Of the patients with FL who achieved CR, all remained in remission at a median follow-up of 24 months. The tolerance appeared acceptable, and no severe cytokine release syndrome (CRS) or immune effector cell–associated neurotoxicity (ICANS) events were observed.

The promising results of these studies led to the pivotal, single-arm phase 2 studies of axi-cel, tisa-cel, and liso-cel in multiple relapsed and refractory FL, the results from which have either resulted in FDA approvals (axi-cel and tisa-cel) or are pending FDA review for approval (liso-cel).

## Axicabtagene ciloleucel

Axi-cel, originally labeled as KTE-C19, is an autologous CAR composed of an extracellular domain targeting CD19, a transmembrane domain, and an intracellular signaling domain composed by a CD28 co-stimulatory molecule.

In March of 2021, the FDA granted accelerated approval to axi-cel for adult patients with R/R FL after two or more lines of systemic therapy based on the results from the ZUMA-5 study. ZUMA-5 was a single-arm, multicenter, phase II trial that included 124 patients with R/R FL requiring treatment as well as 24 patients with marginal zone lymphoma (12). The majority of the patients with FL in ZUMA-5 were stage IV (85%) and with bulky disease (52%). More than half of the patients were identified as POD24 (55%), with a median prior lines of treatment of 3 (range of 2–4) and 63% of patients having had three or more lines of therapy. After lymphodepleting chemotherapy (LDC) with fludarabine and cyclophosphamide, the patients received a single infusion of axi-cel. Among 84 patients with FL eligible for the primary analysis, Jacobson et al. reported a high ORR at 94%, with 79% of patients achieving a CR. Fifty-five percent of the patients with FL treated on the ZUMA-5 trial were identified as POD24, and the outcomes of POD24 patients after receiving axi-cel did not differ significantly from the overall patient population. With a median follow-up of 23.3 months in the original manuscript, the median PFS had not been reached. CRS occurred in 78% patients with FL. Most cases of CRS were grade 1 or 2 [89 (72%)], and grade 3 or worse CRS occurred in eight (6%) patients. For the management of CRS, tocilizumab was administered to 50% and corticosteroids to 18% of the total ZUMA-5 patient population. One grade 5 event of multisystem organ failure leading to death on day 7 was reported in a patient with FL with bulky disease at baseline per GELF criteria. ICANS occurred in 56% of patients with FL, with grade 1 or 2 events in 51 (41%) and grade 3 or 4 events occurring in 19 (15%). No grade 5 neurological events occurred. For management of ICANS, corticosteroids were used in 53 (36%) patients, and tocilizumab was used in nine (6%) of the total ZUMA-5 patient population. Median duration of ICANS was 14 days (IQR of 5–43) in patients with FL, and two patients had ongoing ICANS at the time of publication (one patient with ongoing memory loss and patient with persistent paresthesia).

With additional follow-up at 3 years (median of 40.5 months), Neelapu et al. reported on patients in ZUMA-5 who had exposure to bendamustine within 6 months of LDC had shorter PFS after axi-cel, thought possibly related to the lymphotoxic effects of bendamustine (23). Most recently, Neelapu et al. reported a 4-year follow-up (median of 52.5 months) where the median DOR in patients with FL had lengthened to 55.5 months (24). Patients with a best response of CR had excellent outcomes with median DOR of 60.4 months. Patients that did not achieve CR fared far worse, with a 4.9-month median DOR reported in patients that only achieved a partial response. This longer follow-up led to an improved median PFS of 57.3 months in the patients with FL with an estimated 48-month PFS rate of 53% and a median OS that had still not been reached. Remarkably, only one patient with FL had progressed in interim between the 28 month and 48 month analysis (**Table 1**).

**Table 1:** Comparison of efficacy and safety of CAR T-cell therapy in patients with R/R FL.

Car T-cell product	Reference	Number of patients with R/R FL	ORR (%)	CR (%)	Median PFS [months (mo)]	Median OS	Median DOR Months (mo)	CRS (grade 3+)	ICANS (grade 3+)
Axi-cel	ZUMA-5 (4-year update) (24)	124	94	79	57.3 mo	NR	55 mo	78% (6%)	56% (18%)
Tisa-cel	ELARA (3-year update) (25)	97	86	68	37 mo	NR	NR	49% (0%)	23% (1%)
Liso-cel	TRANSCEND-FL (26)	101	97	94	12 mo, 81%	NR	12 mo, 82%	58% (1%)	15% (2%)

Longer follow-up is needed to assess the curative potential of axi-cel in patients with FL. The ZUMA-22 trial is an ongoing phase 3 randomized study evaluating the benefit of axi-cel compared to standard-of-care therapy for patients with R/R FL. In the absence of available prospective data comparing axi-cel with standard of care (SOC), Ghione et al. compared the results from ZUMA-5 with the International Scholar-5 cohort of patients with R/R FL treated with a third or higher line of SOC at seven different multinational institutions as well data from the phase 2 study of idelalisib in r/r FL (27, 28). This comparative and weighted analysis showed superiority in outcomes related to axi-cel relative to SOC: the median PFS was 57.3 months *vs*. 13.0 months [hazard ratio (HR), 0.27; 95% confidence interval (CI), 0.18–0.40), respectively. Median OS was NR in either study, but, at 48 months, OS was 72.4% in ZUMA-5 compared to 61.4% in SCHOLAR-5. The results strongly suggest the superior efficacy of axi-cel for R/R FL compared to other available SOC therapies.

The activity and safety of axi-cel outside of a clinical trial have been investigated by Jacobson et al. through a CIBMTR registry study where 151 patients with R/R FL underwent treatment with axi-cel and had evaluable assessments post-infusion (29). Forty percent of the patients in this registry study would have been ineligible for ZUMA-5, mainly due to comorbidities. Patients had a median of 4 (range of 1–13) lines of prior therapy. ORR and CR rates were 93% and 84%, respectively, and estimated PFS and OS at 6 months were 88% and 96%, respectively. Grade ≥ 3 CRS and ICANS occurred in 2% and 13% of patents, respectively. The median cumulative CRS resolution was seen by 5 days, and ICANS resolution was resolved on average by day 4. Of the patients that were alive at day 30 (n = 150), 11% had prolonged cytopenias (9% thrombocytopenia and 4% neutropenia). PFS and OS at 6 months were comparable regardless of ZUMA-5 eligibility. The patients that would have met eligibility criteria for ZUMA-5 experienced fewer grade ≥ 3 ICANS (10% *vs*. 16%) and more rapid ICANS resolution (92% *vs*. 71% resolved within 2 weeks). Patients aged ≥ 65 years *vs*. < 65 years were shown to have comparable effectiveness and safety profiles, supporting the safety and efficacy of axi-cel older patients with FL. The CIBMTR registry study confirmed that axi-cel demonstrates effectiveness and safety profiles consistent with those observed in the ZUMA-5 when used in a broader patient population outside of clinical trial.

## Tisagenleucel

Tisa-cel is an autologous CAR T-cell composed of an extracellular domain targeting CD19 like axi-cel but differs in that it is constructed with a 4-1BB co-stimulatory molecule as opposed to CD28. The encouraging efficacy and tolerability of CTL019 reported by Dr. Schuster et al. at the University of Pennsylvania spurred the further clinical development of tisa-cel in FL. The phase II ELARA trial investigated the efficacy of tisa-cel in a larger R/R FL patient population. Ninety-seven patients with FL with at least two lines of prior therapy or who were relapsing after autologous hematopoietic stem cell transplant (HSCT) received a single infusion of tisa-cel following LDC (fludarabine and cyclophosphamide). ORR was reported at 86%, with 69% of the patient population achieving a CR (30). CRS was seen in 49% and ICANS in 4% of patients, but these events were generally low grade; grade 3 or 4 CRS and ICANS were reported in 0% and 1% of patients, respectively. There were no treatment related deaths. On 27 May 2022, the FDA granted accelerated approval to tisa-cel for adult patients with patients with R/R FL after two or more lines of systemic therapy based on the outcomes seen in the ELARA trial. The study was later updated with 3-year follow-up (median follow-up of 41 months), and, at 36 months, 53% of patients remained in CR and the median PFS of the patient population was 37 months (25). Median DOR was NR (**Table 1**). Sixty-three percent of the patients treated on ELARA were identified as POD24, and, within the POD24 subgroup, 36-month PFS was 50% (n = 61) compared with 59% for patients without POD24 (n = 33). Persistence of CAR transgene was observed for up to 1,290 days. The patients that were not POD24 had higher median *in vivo* CAR expansion and longer persistence than patients with POD24.

Salles et al. utilized the data from the ELARA study to perform a comparative effectiveness analysis that matched tisa-cel–treated patients to similar patients in a historical control. The data from the control was utilized to perform a matched adjusted retrospective comparison of patients with R/R FL treated with SOC interventions to similar patients treated with tisa-cel. This analysis showed tisa-cel to be more efficacious than SOC: ORR was 86% for tisa-cel versus 64% for SOC; 12 month PFS 70.5% versus 52%; and 12-month OS was 97% for tisa-cel compared to 72% SOC (31).

In the ELARA study, tisa-cel was administered in the outpatient setting in 18% (17/97) of patients (30). Fowler et al. later evaluated the hospitalization costs and the amount of healthcare resource utilization for the patients with R/R FL undergoing CAR T-cell therapy with tisa-cel comparing inpatient administration versus outpatient administration. Patients infused in the outpatient setting generally had favorable Eastern Cooperative Oncology Group (ECOG) performance status and Follicular Lymphoma International Prognostic Index scores and less bulky disease at baseline (32). For the patients treated in the outpatient setting, 41% did not require hospitalization within 30 days after infusion, and the patients who did ultimately require hospitalization had a shorter average length of stay compared with the patients that received inpatient tisa-cel administration (5 days versus 13 days). Efficacy between the two groups was similar, and the calculated cost of care was reduced for those patients that received treatment in the outpatient setting. These findings supported the premise that some R/R patients can be safely treated with tisa-cel in the outpatient setting, thus reducing hospitalization costs and healthcare resource utilization.

Dickinson et al. performed a match-adjusted indirect comparison (MAIC) of the results reported in the ELARA and ZUMA-5 trials. The results showed that tisa-cel (n = 52), compared with axi-cel (n = 86), had similar ORR (91.2% *vs*. 94.2%; p = 0.58), CR rate (74.0% *vs*. 79.1%; p = 0.60), PFS [HR (95% CI), 0.8 (0.4, 1.9); p = 0.67], and OS [HR (95% CI), 0.5 (0.2, 1.5); p = 0.21] (33). Tisa-cel was associated with more favorable safety outcomes than axi-cel, with lower rates of any grade and grade ≥3 CRS and ICANS seen in patients treated with tisa-cel. After matching, the rates for any grade and grade ≥3 CRS were 33.7% and 6.5% lower (p < 0.01), respectively, and any grade and grade ≥3 ICANS were 47.0% and 15.1% lower (p < 0.001), respectively, in patients infused with tisa-cel *vs*. axi-cel. The proportions of patients who received tocilizumab and corticosteroids for any grade CRS were 35.3% (p < 0.001) and 12.3% lower (p < 0.01), respectively, in patients infused with tisa-cel *vs*. axi-cel.

## Lisocabtagene maraleucel

Liso-cel is also an autologous is a CD19 targeting CAR T-cell with a 4-1BB co-stimulatory molecule with the final product containing CD4:CD8 T cells in a 1:1 ratio. Liso-cel is currently approved by the FDA for second-line or later treatment of DLBCL and is under active investigation for the treatment of R/R FL.

The TRANSCEND FL study is a single-arm, multicenter phase II study where patients with R/R FL who had previously received 1 or 2+ lines of therapy were treated with a single infusion of liso-cel following LDC (fludarabine and cyclophosphamide). The primary analysis of 124 patients treated in the third line was presented with high ORR at 97%, and nearly all of the responding patients achieving a CR (94%) despite being a high-risk patient population where 43% of patients were POD24 (26). With approximately 17 months of median follow-up, 12-month DOR and PFS in patients treated with liso-cel were 81.9% and 80.7%, respectively. CRS occurred in 58% of patients (grade 3, 1%; no grade 4–5) and ICANS in 15% (grade 3, 2%; no grade 4–5). There was one death due to a treatment related adverse event from a grade 5 macrophage activation syndrome (**Table 1**).

## Mosunetuzumab

Mosunetuzumab is a CD20/CD3-bispecific monoclonal antibody that engages endogenous T cells to attack malignant CD20-expressing B cells, showing high response rates and encouraging tolerability in a large phase 2 study. In contrast to the single infusion of CAR T-cell therapy, mosunetuzumab is administered intravenously weekly for the first 3 weeks as part of a double step-up dose and then every 3 weeks for a total of 8 cycles. Nine additional cycles can be administered if complete response is not initially achieved after eight cycles. Budde et al. reported outcomes on 90 patients with median follow-up of 18.3 months; ORR 78% and 60% of patients achieved a CR (11). CRS was the most common adverse event reported at 44% but was predominantly grade 1/2, with only 1% experiencing higher grade CRS. No treatment-related fatal adverse event occurred. Extended 3-year follow-up showed median DOR was 35.9 months, median duration of CR was NR, and the estimated 30-month duration of CR rate was 72.4% (34). Three years after the completion of treatment, 57% of 70 responding patients were alive and had not had disease progression, and the overall median PFS was 24.0 months (**Table 2**).

**Table 2:** CAR T-cell therapy compared to mosunetuzumab.

	Mosunetuzumab	Axi-cel	Tisa-cel	Liso-cel
Trial	Schuster et al. 3-year update (34)	ZUMA-5 (4-year update) (24)	ELARA (3-year update) (25)	TRANSCEND-FL (26)
Number of patients with R/R FL	90	124	97	101
Median prior lines of therapy	3	3	3	3
Median PFS	24 mo (58% at 12 mo)	57 mo (80% at 12 mo)	37 mo (75% at 12 mo)	Median NR at 16 mo (81% at 12 mo)
ORR	78%	94%	86%	97%
CR	60%	79%	68%	94%
CRS (grade 3+)	44% (2%)	78% (6%)	49% (0%)	58% (1%)
ICANS (grade 3+)	5% (0%)	56% (18%)	23% (1%)	15% (2%)

## CAR T cells versus mosunetuzumab

The approvals of CD19 CAR T cells and CD20 bispecifics for multiple relapsed FL are transformative for this high-risk group and based on vastly improved depth and DOR and are expected to improve survival for this group of patients. How to sequence these new therapies in the third line and beyond is not yet established and an area of debate. With a disease like FL, where treatments are not historically curative but patients live for a long time, balancing treatment efficacy and toxicity as well as cost becomes a priority. On the one hand, CD19 CAR T cells represent a one-time treatment with the longest PFS of any available therapies in this space. On the other, it is an expensive and logistically complicated therapy only offered at select centers and does carry risks of higher grade CRS and ICANS compared with the CD20 bispecifics. No randomized trials yet exist to determine which is best for these patients, and so treatment decisions are often made for individual patients, taking into account their disease risk features and their preferences in order to develop an individualized treatment plan.

Nastoupil et al. conducted a MAIC of liso-cel patients from the TRANSCEND FL trial versus published data of mosunetuzumab in patients with 3L+ FL. Their analysis showed liso-cel was associated with higher ORR [odds ratio (OR), 3.78 (95% CI, 1.48−9.67)] and CR [OR, 6.46 (2.85−14.65)], as well as improved PFS [HR, 0.28 (0.16−0.49)] with the results remaining consistent across all scenario analyses (35). Liso-cel was associated with higher incidence of CRS [OR, 1.86 (1.01−3.43)] and ICANS [OR, 2.16 (0.72−6.44)], but liso-cel demonstrated a lower incidence of grade ≥ 3 CRS [OR, 0.45 (0.04−5.13)] and grade 3–4 serious infections [OR, 0.35 (0.12−1.03)] compared with mosunetuzumab. Additionally, liso-cel was associated with overall lower use of steroids for CRS management [OR, 0.14 (0.03−0.65)], but the use of tocilizumab was higher in liso-cel treated patients [OR, 2.27 (0.86−5.99)]. Although imperfect, analyses such as these strongly suggest that logistical considerations and cost effectiveness aside, CD19 CAR T cells with a 4-1BB costimulatory domain are more effective and equally tolerated compared with CD20 bispecifics. Cost analyses have been conflicting, with some data suggesting CAR T-cell therapy is the more cost-effective choice, whereas other data suggesting the opposite (36, 37). At this time, there is no official guidance as to which therapeutic modality (CAR T-cell therapy versus mosunetuzumab) should be sequenced first in patients with R/R FL. With ongoing investigations of moving bispecific antibodies into earlier lines of treatment, both as single agents and in combinations with other treatments, the argument over which treatment to sequence first may soon become irrelevant.

## Future directions

CD19 antigen loss has been reported as a mechanism of resistance for patients that relapse after receiving CD19-directed CAR T-cell therapy. Development of CAR T cells that are directed at other antigens or bispecific CAR T cells with more than one target provides a possible answer for patients with relapses driven from CD19 antigen loss. CD20 CAR T cells and dual targeting of CD20 and CD19 with both bispecifics and CAR T cells are also in development. Shadman et al. reported on a pilot study investigating MB-106, which is a third-generation CD20-directed CAR T-cell constructed with both 4-1BB and CD28 co-stimulatory domains. Three patients with FL were part of the initial 11-patient NHL cohort reported, and two of the three patients with FL achieved a CR, whereas the third patient progressed (38). This was followed by a subsequent report where MB-106 was trialed specifically in 20 patients with R/R FL with a median of 4 prior lines of therapy. ORR was 95%, and the rate of CR was 80%, with overall better responses seen in patients treated at the higher doses of MB-106 (39). Notably, there was one patient on the study who had progressed after previously receiving a CD19-directed CAR, and this patient achieved a CR with MB-106. No grade 3 or 4 CRS or ICANS was observed. Both Shah and Tong et al. have reported separate first in human data on trials of bispecific anti-CD20, anti-CD19 CAR T cells. While these studies only included a small number of patients with R/R FL, they do provide a proof of concept in showing efficacy of these bispecific CAR-T agents in this patient population (40, 41).

Potential limitations of autologous CAR T-cell agents include logistics, product availability manufacturing, and quality consistency. Allogeneic CAR T-cell therapy derived from healthy donors offer an alternative to the available autologous CAR T-cell products and may be able to circumvent these challenges. The ability to infuse treatment to the patient more quickly with these products being available off the shelf and not requiring apheresis and subsequent cell manufacturing may be advantageous in some circumstances, such as patients with very aggressive disease. Locke et al. have reported on safety outcomes of the ALPHA trial, a phase 1 trial exploring the safety and efficacy of an anti-CD19 allogeneic CAR T-cell administered over split doses that utilizes gene editing to control for host lymphocyte rejection. The trial included both R/R DLBCL (n = 61) and FL (n = 26) where 20 patients (23%) experienced CRS, which were low grade except for one (1%) grade 3 event. No graft-versus-host disease or grade ≥3 ICANS occurred (42). These safety data are encouraging, and we will await further reports of efficacy of allogeneic CAR T-cell therapies in patients with R/R FL.

## Expert opinion

CAR T-cell therapies now represent an additional effective tool in the treatment armamentarium of patients with R/R FL. When considering CAR T-cell therapy alongside other approved options for 3L+ FL, the one-time infusion of CAR T cells is unique to the other available options with more protracted treatment schedules, which may have implications for toxicity risks that accumulate over time, like infection. The high rates of responses to CAR T-cell therapy and its ability to produce durable responses in heavily pretreated patients make it appealing and appear to be superior to the SOC in indirect comparisons. The activity of CAR T-cell therapy is particularly encouraging for patients’ refractory to chemoimmunotherapy and POD24 patients who had historically had a poor prognosis with SOC therapies. The side effects overall appear to be acceptable, with low rates of grade 3+ CRS or ICANS, particularly with the 4-1BB CAR T cells’ tisa-cel and liso-cel. The real-world analyses of the efficacy and safety of patients treated with axi-cel outside of clinical trials are reassuring that these treatments can be safely administered to patients commercially with similar efficacy observed, even in patients that would not have qualified for the clinical trials that led to their approval.

With these data in mind, it is our general approach to prioritize CAR T-cell therapy for all patients with 3L+ FL who have a history of POD24 or refractory disease, as well as for patients who are both suitable candidates and for whom a one-time therapy or a therapy with the longest PFS is preferred. This is especially true for younger patients whose life expectancy is anticipated to be shortened by their multiple relapsed FL. We previously referenced reports from patients in ZUMA-5 who had exposure to bendamustine within 6 months of LDC had shorter PFS after axi-cel, and similar concerning findings regarding bendamustine’s negative impact on the outcome of CAR T-cell therapy in DLBCL have been reported (23, 43). Based on these data, we avoid the use of bendamustine within 6 months of leukapheresis for CAR T-cell therapy. Thankfully, this rarely occurs in 3L+ FL given the common use of bendamustine in the frontline and the long median DOR to frontline bendamustine and CD20 monoclonal antibody therapy.

Barriers remain for the widespread utilization of CAR T cell, however, with the high cost of treatment and lack of access to CAR T-cell centers proving to be insurmountable barriers to many patients. Thus, for older patients, for patients with certain medical comorbidities for whom CAR T-cell therapy would be contraindicated, and for lower-risk patients, including those who have enjoyed long remissions from their first- and second line-therapies, and those who prefer a therapy that can be given locally and without hospitalization, we prioritize treatment with mosunetuzumab.

## Conclusion

Patients with R/R FL have a growing number of heterogeneous treatment options. Historically, treatments in the 3L+ setting often involved additional rounds of immunochemotherapy or anti-CD20 monotherapy, lenalidomide, PI3K inhibitors (which subsequently have been withdrawn from the marketplace), as well as HSCT. No consensus on the sequencing of these agents exists at this time. While high response rates to some of these above therapies have been observed, the DOR was usually short and diminished with each subsequent line of therapy (44). Both CD20 bispecifics and CD19 CAR T cells have made significant inroads in the treatment landscape for FL compared to these previous standards.

Data from the most recent follow-up of mosunetuzumab treatment in patients with R/R FL have shown that many responses to this CD20-bispecific also appear durable. It is likely that both bispecific antibodies and CAR T-cell therapies will have significant roles in the future management of R/R FL, but their sequencing remains to be defined. Mosunetuzumab has the benefit of being an off-the-shelf immunotherapy that avoids some of the challenges with logistics and product availability associated with current CAR T-cell therapies, including distance from a CAR T-cell treatment center and the need to relocate there, time to insurance approval, pheresis, manufacturing time, lymphodepleting chemotherapy, and potential hospitalization. The need for LDC also contributes to protracted cytopenias and T-cell lymphopenia in a portion of patients, and the long-term incidence of myeloid dysplasias and risk for T-cell malignancies has not been comprehensively documented and understood (45). No doubt, these risks, once defined, will help shape this debate. However, CAR T-cell therapy is a one-time therapy and offers the longest PFS of any therapy in the multiple relapsed setting, bispecifics included, and, for some patients, this may represent a definitive therapy. Encouragingly, the more typical CAR T-cell side effects of CAR T cells, CRS and ICANS, are lower than those seen in LBCL, particularly with the 4-1BB CAR T cells where these risks seem similar to those seen with mosunetuzumab. How oncologists and patients balance these relative risks and benefits will shape how these therapies are used; by the time this is sorted out, however, the debate is likely to be moot given the likely use of bispecifics in 1L FL, with CAR T cells reserved for select patients in the 2L and 3L settings. Regardless of sequencing preferences, we are better off for having a multitude of options for patients who need them.

## Author contributions

RJ: Conceptualization, Investigation, Writing – original draft, Writing – review & editing. CJ: Writing – review & editing.
